# Supplementary material for: Association between oral microbiome and seven types of cancers in East Asian population: a two-sample Mendelian randomization analysis
Source: Front Mol Biosci. 2023 Nov 21;10:1327893. doi: 10.3389/fmolb.2023.1327893 (PMC10702768; doi:10.3389/fmolb.2023.1327893)
Supplement: Supplementary file 1 [file DataSheet1.ZIP › Supplementary Materials/MR plots of saliva/saliva═╝/Lung Cancer/pheno.2655_to_lung cancer_forest.pdf]

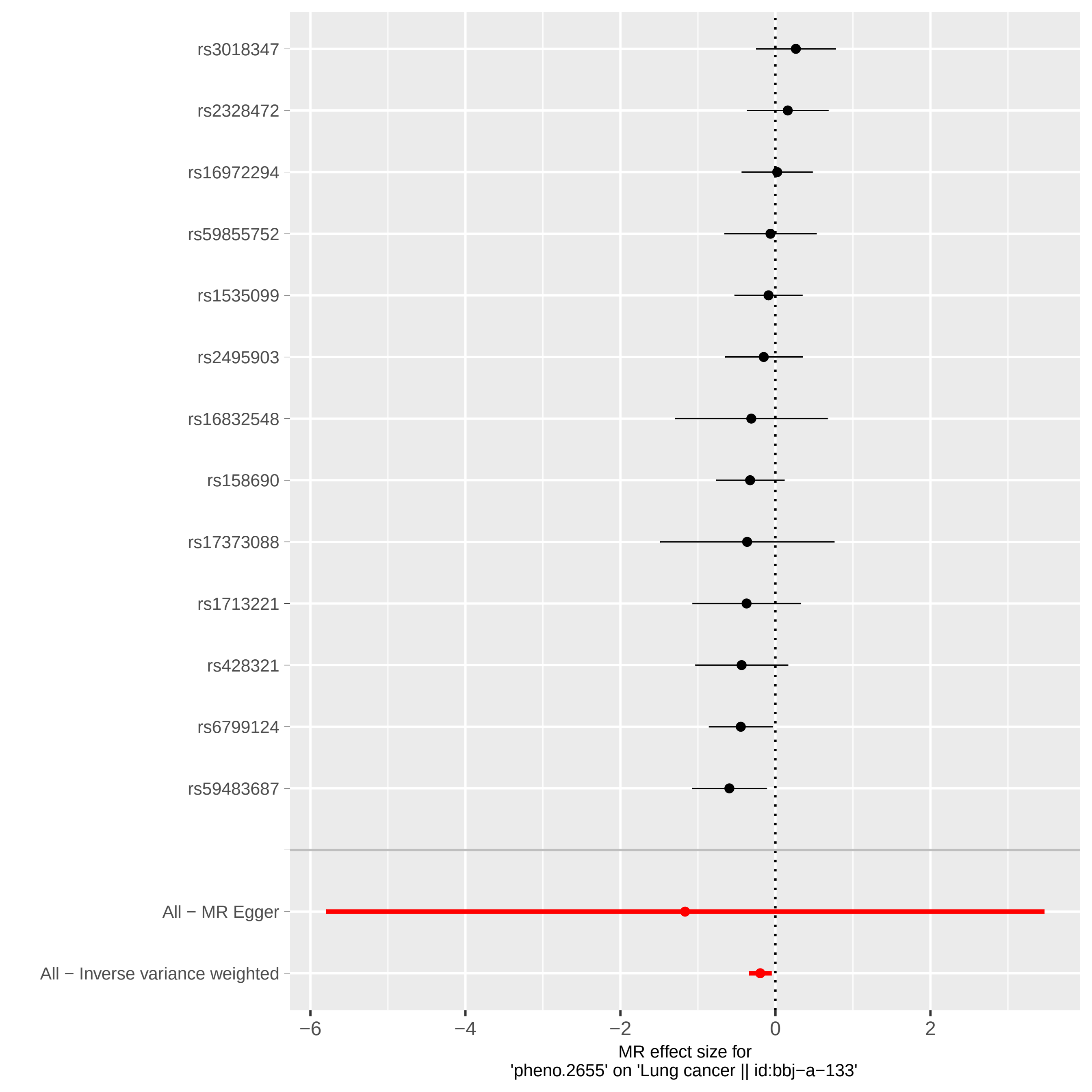
rs3018347
rs2328472
rs16972294
rs59855752
rs1535099
rs2495903
rs16832548
rs158690
rs17373088
rs1713221
rs428321
rs6799124
rs59483687
All − MR Egger
All − Inverse variance weighted
−6
−4
−2
0
2
MR effect size for
'pheno.2655' on 'Lung cancer || id:bbj−a−133'
